# Does charge-free screening improve detection of gestational diabetes in women from deprived areas: a cross-sectional study

**DOI:** 10.1186/s12884-016-1060-3

**Published:** 2016-09-09

**Authors:** Andreas Beyerlein, Daniela Koller, Anette-Gabriele Ziegler, Nicholas Lack, Werner Maier

**Affiliations:** 1 Institute of Diabetes Research, Helmholtz Zentrum München, Neuherberg, Germany; 2 Forschergruppe Diabetes der Technischen Universität München, Munich, Germany; 3 Department of Health Services Management, Munich School of Management, Ludwig-Maximilians-Universität München, Munich, Germany; 4 Forschergruppe Diabetes e.V. am Helmholtz Zentrum München, Munich, Germany; 5 German Center for Diabetes Research (DZD), Partner, Neuherberg, Germany; 6 German Bavarian Quality Assurance Institute for Medical Care, Munich, Germany; 7 Institute of Health Economics and Health Care Management, Helmholtz Zentrum München, Neuherberg, Germany

**Keywords:** Gestational diabetes mellitus, Area level deprivation, Bavarian index of multiple deprivation, Charge-free screening

## Abstract

**Background:**

Gestational diabetes mellitus (GDM) occurs in 2–6 % of all pregnancies. We investigated whether area level deprivation is associated with a higher risk for GDM and whether GDM detection rates in deprived regions changed after the introduction of charge-free GDM screening in Germany in 2012.

**Methods:**

We analyzed population-based data from Bavaria, Germany, comprising *n* = 587,621 deliveries in obstetric units between 2008 and 2014. Area level deprivation was assessed municipality-based using the Bavarian Index of Multiple Deprivation (BIMD), divided into quintiles and assigned to each mother based on her residential address. We estimated annual odds ratios (ORs) for GDM diagnosis by BIMD quintile with adjustment for maternal obesity, maternal age, migration background and single mother status.

**Results:**

Women from the most deprived regions were less likely to be diagnosed with GDM before introduction of charge-free GDM screening (OR = 0.76 [95 % confidence interval: 0.66, 0.86] compared to least deprived areas), in 2008. In contrast, high area level deprivation was associated with significantly increased risk of GDM diagnosis in 2013 (OR [95 % confidence interval] = 1.15 [1.02, 1.29]). The OR was also elevated, although not significantly, in 2014 (OR [95 % confidence interval] = 1.05 [0.93, 1.18]).

**Conclusions:**

The prevalence of GDM seems to have been underreported in women from highly deprived areas before introduction of the charge-free GDM screening in Germany. In fact, women living in deprived regions seem to have an increased risk for GDM and may profit from access to charge-free GDM screening.

## Background

Gestational diabetes mellitus (GDM) occurs in 2–6 % of all pregnancies in industrialized countries [1] with an increasing prevalence in recent years [2, 3]. In 2010, the World Health Organization (WHO) presented new diagnostic criteria for the classification of GDM [4] which Germany adopted in 2011. Based on the new criteria GDM was diagnosed if at least one of the values for fasting, 1-h, and 2-h plasma glucose concentration as measured in a 75-g oral glucose tolerance test exceeded diagnostic thresholds, while at least two elevated values were required for GDM diagnosis before 2011. Apart from that, every pregnant woman in Germany without pre-existing diabetes has been offered a charge-free GDM screening since 2012, whereas formerly the optional test had to be paid for by the mother.

Maternal obesity is the major risk factor for GDM [5] but there are also other potential determinants such as gestational weight gain, maternal age, parity, family history of diabetes, ethnicity or life-style habits [6–10]. At an area level, it was reported that living in neighbourhoods with a high prevalence of fast food restaurants was significantly associated with an increased risk of GDM [11]. Maternal socioeconomic status (SES) might be another potential risk factor. In general, SES can be assessed either at individual level (e. g. based on educational level) or from area-based socioeconomic measures such as deprivation indices. There have been a number of studies on the association between individual level SES and GDM, yielding inconsistent results [12]. The association of area level deprivation and GDM has been rarely investigated, however, and the sparse literature provided rather inconsistent results. While neighbourhood deprivation was not found to be associated with GDM risk in data from Southwest England, higher glucose values at GDM diagnosis were observed in Canadian women living in highly deprived areas [13, 14].

Interestingly, high area level deprivation has already been found to be associated with increased risk for obesity [15] and type 2 diabetes [16, 17], particularly in women [18].

Here, we had the opportunity to analyze a large population-based dataset from Bavaria, Germany, which allowed investigating the association between area level deprivation and GDM risk. Additionally, we were able to assess the impact of the introduction of a charge-free GDM screening [19] on this association.

## Methods

Data were extracted from a standard dataset regularly collected for national benchmarking of obstetric units in terms of clinical performance. The BAQ (Bayerische Arbeitsgemeinschaft für Qualitätssicherung - Bavarian Institute for Quality Assurance in hospital care) conducts corresponding regional evaluations for Bavaria, Germany. Data are transferred electronically to the BAQ office after personally identifying characteristics have been removed and replaced by an anonymous unique reference number. Amongst others, these data contain information on maternal age, weight and height, postal code of the mother’s residential address, GDM, and pre-gestational diabetes as previously described [20, 21]. GDM was diagnosed by the women’s gynaecologists who are assumed to follow the official guidelines from the German Diabetes Association. These guidelines were revised in 2011 following the WHO’s new diagnostic criteria [4]. Information on maternal weight and gestational age at first prenatal care visit was extracted by midwives and nurses from the mother’s pregnancy booklet (issued to every pregnant woman in Germany for complete documentation of all antenatal care visits) and augmented with additional information upon hospital admission. Mother’s height and weight recorded at first visit (median gestational age: 8 completed weeks) were used to derive her body mass index (BMI). The BAQ data do not contain variables on individual level SES apart from fairly rough surrogates such as smoking during pregnancy or migration background.

We assessed area level deprivation using the Bavarian Index of Multiple Deprivation (BIMD) [22] derived from official statistics and methods used in the UK for assessment of regional deprivation [23]. The BIMD including seven subdomains of deprivation (income, employment, education, municipal revenue, social capital, environment, security) was computed for all 2,056 Bavarian municipalities. We categorized the municipalities by BIMD quintiles, with the first quintile designating the least deprived and the fifth quintile the most deprived municipalities. Assignment of BIMD quintile to each mother was done based on postal code of her residential address.

According to current German maternity guidelines established in 2012, every pregnant woman without pre-existing diabetes is offered a glucose-challenge test between the 24^th^ and 27^th^ gestational week. In a second step, women with increased glucose values in the glucose-challenge test will be tested by an oral glucose tolerance test for diagnosis of GDM. The charges of this two-step procedure are now fully covered by the women’s health insurance, whereas formerly the test had to be paid for by the mother. To compare periods before and after its introduction we considered the data of all deliveries in Bavarian obstetric units from 2008 to 2014 (*n* = 645,774). Of these, 4,564 women with known pre-gestational diabetes were excluded. The BIMD could not be assigned to a further 53,589 women because the postal codes of their residential addresses referred to a location outside Bavaria or were missing, thus leaving a final sample size of *n* = 587,621. We used logistic regression to estimate odds ratios (ORs) and corresponding 95 % confidence intervals (CIs) with GDM as the outcome variable and BIMD quintiles as the explanatory variable (lowest deprivation as reference) separately for each year from 2008 to 2014. All models were calculated both crude and adjusted for maternal obesity (BMI ≥ 30 kg/m^2^) at first visit, maternal age (<35 compared to ≥ 35 years), migration background and single mother status, as these were significant predictors in unadjusted analyses. Likewise we assessed associations between GDM and all subdomains of the BIMD (as a continuous variable).

Statistical analyses were performed with SAS (version 9.3, SAS Institute Inc., Cary, NC, USA) and R (version 3.0.3, https://cran.r-project.org).

## Results

The proportion of women with a diagnosis of GDM increased from 3.4 % in 2008 to 4.0 % in 2014 (Table 1), resulting in a relative increase in prevalence of about 17 %. Maternal obesity, age, migration background and single mother status were significantly associated with GDM in both 2008 and 2014 (p < 0.05), while smoking during pregnancy and parity (multiparous compared to primiparous) were not (data not shown). In contrast to GDM, the relative increase in prevalence for any of these factors was 11 % (as observed for obesity) or lower.

**Table 1:** Description of the study population in 2008 and 2014

	Year 2008 (*n* = 81,129)	Year 2014 (*n* = 92,589)
Gestational diabetes mellitus	*n* = 2,745 (3.4 %)	*n* = 3,682 (4.0 %)
Obese (BMI > 30 kg/m^2^)	*n* = 8,819 (10.9 %)	*n* = 11,169 (12.1 %)
Age > 35 years	*n* = 21,163 (26.1 %)	*n* = 25,615 (27.7 %)
Migration background	*n* = 14,401 (17.8 %)	*n* = 17,772 (19.2 %)
Single mother	*n* = 7,731 (9.9 %)	*n* = 6,864 (8.2 %)
Multiparous woman	*n* = 40,635 (50.1 %)	*n* = 45,219 (48.8 %)
Smoking during pregnancy	*n* = 5,457 (6.7 %)	*n* = 4,262 (4.6 %)
BIMD quintile 1 (least deprived)	*n* = 10,306 (12.7 %)	*n* = 12,716 (13.7 %)
BIMD quintile 2	*n* = 9,679 (11.9 %)	*n* = 10,329 (11.2 %)
BIMD quintile 3	*n* = 23,948 (29.5 %)	*n* = 27,831 (30.1 %)
BIMD quintile 4	*n* = 13,198 (16.3 %)	*n* = 14,829 (16.0 %)
BIMD quintile 5 (most deprived)	*n* = 23,998 (29.6 %)	*n* = 26,884 (29.0 %)

*BIMD* Bavarian Index of Multiple Deprivation

**Fig. 1:**
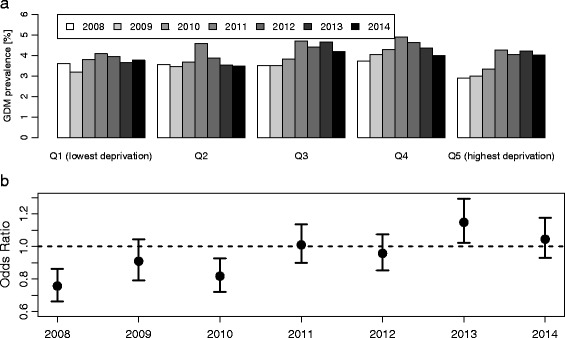
Yearly rates of gestational diabetes mellitus (GDM) diagnoses from 2008 to 2014 (numbers of pregnancies without pre-gestational diabetes in brackets) in Bavaria, Germany, by deprivation quintiles (Q1-Q5, plot a), and yearly odds ratios with corresponding 95 % confidence intervals for Q5 compared to reference Q1 (plot b, all estimates adjusted for maternal obesity, age, migration background and single mother status). In 2011, the criteria for GDM diagnosis were revised in Germany following WHO recommendations. In 2012, charge-free GDM screening, following a two-step procedure, was established in Germany

The rate of GDM diagnosis increased in 2011, when the new definition was established in Germany, in all BIMD quintiles and dropped slightly in 2012 when the universal two-step GDM screening was introduced (Fig. 1). However, the GDM prevalence remained at a considerably higher level compared to before 2011 in women from deprived areas, but not in women from regions with low level deprivation. Women from the most deprived areas were less likely to be diagnosed with GDM in 2008 (OR [95 % CI] = 0.76 [0.66, 0.86] for the fifth quintile compared to the first quintile in adjusted analyses, Table 2). In contrast, a higher area level deprivation was significantly associated with increased risk of GDM diagnosis in 2013 (OR [95 % CI] = 1.15 [1.02, 1.29] for the fifth quintile compared to the first quintile). The OR was also elevated, although not significantly, in 2014 (OR [95 % CI] = 1.05 [0.93, 1.18]). Further analyses suggested that these trends over time were at least partly based on changes in areas with low employment rates, as employment deprivation was the only BIMD subdomain associated with both lower GDM risk by trend in 2008 (OR [95 % CI] = 0.98 [0.94, 1.02]) and significantly higher risk in 2014 (OR [95 % CI] = 1.05 [1.02, 1.09], Table 3).

**Table 2:** Odds ratios [95 % confidence intervals] for gestational diabetes mellitus by Bavarian Index of Multiple Deprivation (BIMD) quintile in 2008 and 2014 (based on *n* = 81,129 and *n* = 92,589 observations, respectively) as categorical variables, and by maternal obesity, age, migration background and single mother status, both crude and mutually adjusted

BIMD	2008, crude	2008, adjusted	2014, crude	2014, adjusted
Quintile 1 (least deprived)	Reference	Reference	Reference	Reference
Quintile 2	0.99 [0.85, 1.15]	0.99 [0.85, 1.15]	0.92 [0.80, 1.06]	0.93 [0.81, 1.08]
Quintile 3	0.97 [0.86, 1.10]	0.92 [0.81, 1.04]	**1.12 [1.001, 1.24]**	1.07 [0.95, 1.20]
Quintile 4	1.04 [0.90, 1.19]	1.01 [0.88, 1.16]	1.06 [0.94, 1.20]	1.09 [0.96, 1.24]
Quintile 5 (most deprived)	**0.79 [0.70, 0.90]**	**0.76 [0.66, 0.86]**	1.07 [0.96, 1.19]	1.05 [0.93, 1.18]
Maternal obesity	**3.07 [2.81, 3.35]**	**3.19 [2.92, 3.49]**	**3.53 [3.28, 3.80]**	**3.71 [3.43, 4.00]**
Maternal age ≥35 years	**1.54 [1.42, 1.67]**	**1.56 [1.44, 1.69]**	**1.64 [1.53, 1.75]**	**1.69 [1.57, 1.82]**
Migration background	**1.44 [1.32, 1.56]**	**1.52 [1.39, 1.67]**	**1.60 [1.49, 1.71]**	**1.68 [1.55, 1.82]**
Single mother status	**1.56 [1.34, 1.81]**	**1.46 [1.25, 1.71]**	**1.31 [1.14, 1.50]**	**1.27 [1.10, 1.46]**

Significant associations (*p* < 0.05) are shown in bold face

**Table 3:** Odds ratios [95 % confidence intervals] for gestational diabetes mellitus by Bavarian Index of Multiple Deprivation (BIMD) domains in 2008 and 2014 (based on *n* = 81,129 and *n* = 92,589 observations, respectively), both crude and adjusted for maternal obesity, age, migration background and single mother status

BIMD domain	2008, crude	2008, adjusted	2014, crude	2014, adjusted
Income deprivation	**0.84 [0.80, 0.87]**	**0.84 [0.80, 0.88]**	**0.91 [0.88, 0.95]**	**0.92 [0.89, 0.96]**
Employment deprivation	1.00 [0.96, 1.04]	0.98 [0.94, 1.02]	**1.07 [1.03, 1.10]**	**1.05 [1.02, 1.09]**
Educational deprivation	**0.88 [0.84, 0.91]**	**0.88 [0.84, 0.91]**	0.97 [0.94, 1.003]	0.98 [0.94, 1.01]
Municipal revenue deprivation	**0.92 [0.88, 0.96]**	**0.93 [0.89, 0.97]**	**0.94 [0.91, 0.97]**	**0.96 [0.93, 0.999]**
Social capital deprivation	**0.87 [0.83, 0.90]**	**0.88 [0.84, 0.91]**	**0.97 [0.93, 0.999]**	0.99 [0.95, 1.02]
Environment deprivation	**1.10 [1.06, 1.14]**	**1.08 [1.03, 1.12]**	**1.11 [1.08, 1.15]**	**1.08 [1.04, 1.12]**
Security deprivation	**1.09 [1.05, 1.13]**	**1.08 [1.04, 1.12]**	**1.06 [1.03, 1.10]**	**1.06 [1.02, 1.10]**

Significant associations (*p* < 0.05) are shown in bold face

## Discussion

Our data show that area level deprivation is associated with GDM prevalence in a subtle way: Women from highly deprived areas were less likely to be diagnosed with GDM than women from less deprived regions before introduction of the charge-free GDM screening, but vice versa after 2012.

Hence, it appears likely that GDM was largely underreported in women from deprived areas before 2012. We therefore assume that living in deprived regions, especially with high unemployment rates, is indeed associated with a slightly increased risk for GDM as observed in our data from 2013 (significantly) and 2014 (not significantly). This is in accordance with previous studies related to type 2 diabetes [16–18]. Our findings further indicate that particularly women from deprived areas may profit from charge-free GDM screening. Although the screening fees were only about 10–25 €, we assume that this may already have constituted a financial barrier for an unemployed single woman in Germany receiving state benefits of only 364 € (“Hartz IV”) monthly in 2011. This would agree well with findings from other studies, indicating that especially people with low SES tended to avoid or delay physician visits after a practice charge for physicians (10 € per calendar quarter) was introduced in Germany in 2004 [24], and that financial barriers may be relevant for participation in colorectal cancer screening [25, 26].

In general, the trends of GDM prevalence in our data appear plausible, although they could only partly be explained by concomitant increases in the prevalence of risk factors such as maternal obesity or increased maternal age. Indeed, we observed an increase in the prevalence of both risk factors in our data, which is in line with long-term temporal trends in Germany [27, 28]. However, we think that it appears likely that the marked increase in GDM prevalence in 2011 in our data was due to the revision of the GDM diagnosis criteria in Germany in the same year, which may have particularly improved the detection of milder GDM cases (as only one instead of two elevated blood glucose values was deemed sufficient for GDM diagnosis). The lower prevalence in the following years may be due to introduction of the glucose-challenge pre-test in 2012, which is known to miss about 20 % of all GDM cases [29]. Interestingly, we observed almost no such decrease in prevalence in women from mostly deprived areas, possibly indicating that the introduction of charge-free screening levelled out the numbers missed by pre-testing in this subgroup. We are, however, hesitant to overinterpret GDM prevalence rates from 2011 and 2012, as changes introduced in these years may not have been immediately adopted by physicians [30] thus possibly leading to regional differences in this respect. Unfortunately, we are not aware of any other study reporting temporal trends of GDM prevalence in Germany before and after 2011–2012 to support our assumptions.

A major strength of the presented results is seen in the large number of pregnancies available for analysis. However, the data were collected for purposes not related to the study hypothesis. Data quality is high as completeness of the data is monitored annually across obstetric units as an integral part of benchmarking health-care provision: The BAQ, providing the data for our study, is part of an established national program to generate annual statistics depicting an individual obstetric units’ deviations from national targets. This program has been run within all federal states over the last three decades. Quality assurance data from the UK and the USA have similarly been analysed in other studies on determinants of pregnancy outcomes [31–35].

Unfortunately, the BAQ data do not contain a valid indicator of the mother’s SES so that we were not able to distinguish between the associations of individual and area level deprivation on GDM prevalence. It should further be noted that the BIMD quintiles were calculated based on the number of municipalities, as opposed to the population size of each municipality. Therefore, the proportions of pregnant women were not equally distributed across BIMD quintile. This was similar to previous studies [17, 18], however, and we do not think that this might constitute a potential source of severe bias.

In summary, our findings indicate that women living in highly deprived areas have a higher risk of developing GDM, and that even moderate charges may constitute an obstacle for participation in GDM screening. Thus, access to charge-free GDM screening and to appropriate treatment may be an important step towards more equality in pregnancy-related health care.

## Abbreviations

BAQ: Bayerische Arbeitsgemeinschaft für Qualitätssicherung
BIMD: Bavarian index of multiple deprivation
BMI: Body mass index
CI: Confidence interval
GDM: Gestational diabetes mellitus
OR: Odds ratio
SES: Socioeconomic status
WHO: World Health Organization
